# Synergistic Preservation of Fresh Pork: Coupling Electrostatic Field and Packaging During Controlled Freezing-Point Storage

**DOI:** 10.3390/foods14223890

**Published:** 2025-11-14

**Authors:** Wenxin Wang, Le Liu, Ming Tian, Xiaotong Sun, Ruixin Shi, Jiarui Li, Debao Wang, Qingfeng Yang, Dequan Zhang, Chengli Hou

**Affiliations:** 1 Institute of Food Science and Technology, Chinese Academy of Agricultural Sciences, Key Laboratory of Agro-Products Quality and Safety Control in Storage and Transport Process, Ministry of Agriculture and Rural Affairs, Beijing 100193, China; wangwenxinedu@163.com (W.W.); liule1017@126.com (L.L.); 13080204653@163.com (M.T.); jasmine5689@163.com (X.S.); ruixinshi@126.com (R.S.); 82101232160@caas.cn (J.L.); wangdebao@caas.cn (D.W.); dequan_zhang0118@126.com (D.Z.); 2 Institute of Western Agriculture, Chinese Academy of Agricultural Sciences, Changji 831100, China

**Keywords:** electrostatic field, fresh pork, controlled freezing-point storage, packaging, microbial diversity

## Abstract

To address spoilage and quality deterioration, this study evaluated the synergistic effects of the electrostatic field (EF) combined with packaging (polyethylene, PP; vacuum, VP; modified atmosphere, MAP) on the preservation of *Yorkshire* pork (hind leg) during controlled freezing-point storage (−2.0 ± 0.5 °C) for 32 days. The results showed that EF treatment significantly enhanced the water-holding capacity of PP-packaged pork, reducing storage loss by approximately 37.89% by day 32 (*p* < 0.05), and inhibited microbial growth, maintaining total viable counts below 6.00 log_10_ (CFU/g) (*p* < 0.05). EF also reduced the relative abundance of spoilage organisms such as *Pseudomonas*. A synergistic effect between EF and VP/MAP was observed in the optimization of the microbial community structure. Spearman correlation analysis revealed that *Pseudomonas* abundance was positively correlated with TVB-N and storage loss, linking it mechanistically to quality deterioration. Furthermore, VP and MAP alone were superior in delaying lipid oxidation (TBARS < 0.5 mg MDA/kg) and maintaining color stability. This study provides key process parameters and a theoretical basis for applying EF-coupled packaging in the industrial cold chain.

## 1. Introduction

Pork is one of the most consumed meats globally. However, due to its rich nutritional content, including high levels of water, protein and fat, pork is highly susceptible to spoilage during transportation, storage, and retail, driven by enzymatic and microbial activity [1]. Meat spoilage is a complex process, primarily instigated by microbial proliferation and metabolism, leading to the subsequent accumulation of spoilage compounds. The rate of spoilage is influenced by numerous factors, including hygiene conditions in the processing environment, packaging type, storage temperature, and meat pH value [2,3]. Among these, temperature and packaging methods are considered critical determinants of pork shelf-life [4,5]. To extend the shelf-life of pork, various preservation strategies have been developed.

In recent years, the combination of controlled freezing-point storage with different packaging technologies has garnered significant attention due to its cost-effectiveness, environmental sustainability, safety, and effectiveness in preserving meat quality during storage [6]. With further research, electrostatic field (EF)-assisted controlled freezing-point storage for meat and other solid foods has demonstrated considerable potential [7,8,9]. As a non-thermal, non-contact, and environmentally friendly physical sterilization technology, the EF, particularly the high-voltage EF (HVEF), exhibits strong bactericidal potential. This is attributed to its ability to disrupt microbial cell membranes via electroporation and induce air ionization, generating secondary bactericidal factors such as reactive oxygen species and ozone [7,10,11]. Previous studies have shown that it can significantly reduce the relative abundance and activity of pathogenic or spoilage microorganisms in fruits, vegetables, aquatic products, and certain meat systems [12,13,14,15]. However, systematic research, specifically on chilled pork, remains relatively scarce.

Common pork packaging methods include polyethylene packaging (PP), vacuum packaging (VP), and modified atmosphere packaging (MAP) [3,16,17]. The oxygen permeability of PP packaging maintains meat color during short-term storage. However, this permeability can lead to accelerated lipid oxidation in meat products during extended storage. In contrast, VP packaging inhibits the growth of aerobic microorganisms by removing oxygen [18,19]. MAP delays oxidation and the growth of anaerobic/aerobic microorganisms by adjusting the oxygen concentration and gas composition within the package [17,20]. Although HVEF and packaging strategies each have the potential to extend meat shelf-life, the synergistic mechanisms of their combination have not been fully elucidated. Specifically, the antibacterial effect of HVEF is highly dependent on the combination of voltage intensity and treatment time [10,21]; excessive voltage or improper treatment may lead to surface charring or sensory damage, while insufficient treatment may not effectively penetrate deeply embedded contaminants [22,23]. Existing studies have indicated a trade-off between antibacterial efficiency and energy efficiency in intermittent and continuous electric field treatments: continuous EF exhibits stronger antibacterial activity in the short term, but intermittent EF is more advantageous in terms of energy consumption and economics, which is closely related to the electric field intensity [7,13,21]. Therefore, in practical cold chain scenarios, it is necessary to comprehensively consider antibacterial effects, energy consumption, packaging strategies, and superchilled conditions to establish an industrialized and optimized solution.

Based on the above background, this study aims to investigate the impact of coupling an EF with different packaging methods on the preservation of pork under controlled freezing-point storage in order to meet the practical requirements of pilot-scale cold-chain operations. The study will systematically compare PP, VP, and MAP systems with respect to their effects on the temporal dynamics of total viable counts (TVCs), total volatile basic nitrogen (TVB-N), thiobarbituric acid reactive substances (TBARSs), textural properties, and water-holding capacity (WHC) during controlled freezing-point storage of pork, and will integrate microbial diversity analyses to reveal changes in community structure and quality characteristics. The findings aim to provide the necessary process parameters and mechanistic insights to enable the industrial application of EF for the controlled freezing-point storage of pork, aiming for a safe, scalable, chemical-free, and low-energy preservation solution.

## 2. Materials and Methods

### 2.1. Materials

The present study utilized pork hind legs obtained from six *Yorkshire* gilts (approximately 6.5 months old, 126–129 kg), which were provided by Beijing Ershang Meat Food Group Co., Ltd. (Beijing, China), after 24 h of post-slaughter chilling. Visible fat and connective tissue were removed from each hind leg, and the meat was cut into 25 uniform pieces (150 ± 5 g) per leg, yielding a total of 150 samples. The meat pieces were randomly assigned to three packaging treatments: (1) PP; (2) VP; and (3) MAP (80% O_2_ and 20% CO_2_). Each packaging group was further divided randomly into two subgroups: one subjected to an EF treatment (designated as EPP, EVP, and EMAP, respectively) and the other without electrostatic treatment (designated as PP, VP, and MAP, respectively). All treatments were carried out at −2 ± 0.5 °C.

The oxygen transmission rates of the packaging materials were as follows: PP (polyethylene, supplied by Tuopu Daily Chemicals Co., Ltd., Ningbo, China): 20,879.72 cm^3^/m^2^·24 h·0.1 MPa; VP (polyamide/ethylene vinyl alcohol copolymer/polyethylene, supplied by Sunrise Material Co., Ltd., Jiangyin, China): 2.50 cm^3^/m^2^·24 h·0.1 MPa; MAP (polyamide/polyethylene film, supplied by Sunrise Material Co., Ltd., Jiangyin, China): 17.25 cm^3^/m^2^·24 h·0.1 MPa.

During storage (0, 8, 16, 24, and 32 days), the gas composition inside the MAP packages was monitored and confirmed to remain stable. Samples were collected for subsequent analysis, with six independent biological replicates (n = 6) performed for each specified condition.

### 2.2. Cold Storage with the EF

**Figure 1:**
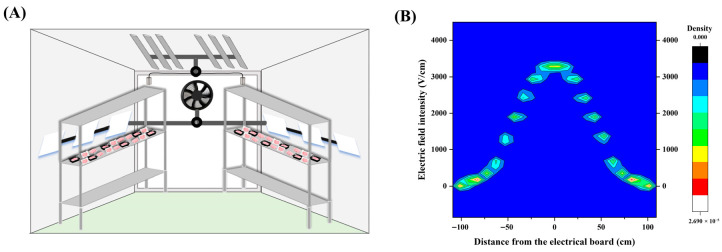
(**A**) Schematic illustration of the EF cold storage chamber; (**B**) distribution of EF strength inside the cold storage chamber.

The cold storage unit measured 6 × 2 × 2.5 m and incorporated an EF generated by six unipolar discharge plates connected in series. It was jointly developed by the Institute of Food Science and Technology of the Chinese Academy of Agricultural Sciences and Suzhou SVK Special Equipment Manufacturing Co., Ltd. (Suzhou, China). The EF system operated at an input voltage of 220 V, producing an output voltage of 4.0 kV at a frequency of 50 Hz (Figure 1A). The EF strength distribution within the cold storage unit is shown in Figure 1B, demonstrating a gradual decrease in field strength with increasing distance from the discharge plates. To ensure consistent experimental conditions, samples were positioned within a region where the EF strength was stable, ranging from 3.2 to 3.3 kV/cm.

### 2.3. WHC

#### 2.3.1. Storage Loss

Accurately measure the weight of the sample before (M0) and after storage (M1). The storage loss was calculated using Equation (1):

(1) $$\mathrm{Storage}\;\mathrm{loss}\;(\%)=\frac{M0\;-\;M1}{M0}\times 100$$

#### 2.3.2. Cooking Loss

Weigh 30 ± 0.5 g of the meat sample (M2) and place it in a 71 °C constant temperature water bath. Cook for 30 min, then absorb the surface moisture of the meat sample and weigh it again (M3) [24]. Calculate the cooking loss according to Equation (2):

(2) $$\mathrm{Cooking}\;\mathrm{loss}\;(\%)=\frac{M2\;-\;M3}{M2}\times 100$$

#### 2.3.3. Centrifuging Loss

Each 2 ± 0.05 g thawed sample was centrifuged (8000× *g*, 4 °C) for 30 min after being wrapped in filter paper. The centrifuging loss of pork was calculated using Equation (3) [21]:

(3) $$\mathrm{Centrifuging}\;\mathrm{loss}\;(\%)=\frac{M4\;-\;M5}{M4}\times 100$$

where M4 represents the weight of the sample before centrifugation, and M5 denotes its weight after centrifugation.

#### 2.3.4. Magnetic Resonance Imaging (MRI)

Proton density imaging was conducted on a NIUMAG I20-040H-I NMR system (Suzhou, China). Samples (1 × 1 × 2 cm) were centered in the magnetic field and imaged with TR = 2000 ms, 4 repetitions, T1 = 20 ms, and TE = 20 ms. Pseudo colored images were then generated using NIUMAG’s evaluation software (V1.0, Shanghai, China) [9].

### 2.4. Color

Using a calibrated Konica Minolta CM-600D portable chroma meter (Tokyo, Japan) [25], pork sample color (L*, a*, b*) was measured. Samples were equilibrated at 25 ± 1 °C for 10 min after film removal, and six randomized surface points were analyzed per sample, with six biological replicates per treatment group.

### 2.5. TBARS

To evaluate lipid oxidation, TBARS content was analyzed using malondialdehyde bis (formaldehyde) as a reference compound (0–2 ppm calibration range) according to previous methods, and the value was calculated as MDA equivalent (mg/kg sample) [26].

### 2.6. pH Value

Briefly, a pork sample accurately weighed at 5.00 ± 0.02 g was homogenized in 50 mL of a 0.10 mol/L KCl solution, filtered, and the filtrate’s pH was subsequently determined with a calibrated pH meter, with the mean value recorded for further analysis [26].

### 2.7. Shear Force

The meat samples used for determining cooking loss were cut into small pieces measuring 3.0 cm × 1.0 cm × 1.0 cm. Shear force was measured using a Stable Micro Systems TA-XT plus^®^ texture analyzer (Surrey, UK) with an HDP/BSW probe, ensuring muscle fiber orientation was perpendicular to the cutting surface. Test parameters included a pre-test speed of 2 mm/s, a test speed of 2 mm/s, and a post-test speed of 5 mm/s.

### 2.8. Microbial Analysis

A 5.00 ± 0.20 g of pork was homogenized with 45 mL of sterile saline. The resulting homogenate was processed using a flapping homogenizer for 2 min, followed by serial dilution in sterile saline at a 1:10 (*v*/*v*) ratio. After mixing 1 mL of serial dilution with Plate Count Agar and incubating at 37 °C for 48 h, the results were represented as log10 (CFU/g) (lg(CFU/g)) sample [27].

### 2.9. TVB-N

A 5.00 ± 0.05 g pork sample was homogenized with 25 mL of distilled water and left to stand for 30 min before filtering. Then, 10 mL of the filtrate was mixed with 5 mL of MgO solution (10.00 g/L) and distilled for 5 min using a Kjeldahl distillation unit. The distillate was collected in 10 mL of boric acid solution with five drops of indicator and titrated with 0.01 mol/L HCl. The TVB-N content was expressed as mg/100 g of pork [26].

### 2.10. Microbial Community Characterization

Total genomic DNA was extracted from samples using the FastDNA™ Spin Kit for Soil, 6560-200 (MP Biomedicals, Southern, CA, USA) . The V3-V4 hypervariable region of the 16S rRNA gene was amplified by PCR using the barcoded forward primer 338F (5′-ACTCCTACGGGAGGCAGCAG-3′) and reverse primer 806R (5′-GGACTACHVGGGTWTCTAAT-3′). PCR amplification was performed in a 20 μL reaction volume with an initial denaturation at 95 °C for 3 min, followed by 27 cycles. PCR products were recovered from a 2% agarose gel and purified using a DNA gel extraction kit. Amplicons were then identified by 1% agarose gel electrophoresis and subsequently purified using the AxyPrepDNA PCR Clean-up Kit (Axygen Biosciences, Union City, CA, USA). Libraries were constructed from the purified PCR products using the NEXTFLEX Rapid DNA-Seq Kit and sequenced on the Illumina Nextseq2000 platform (Majorbio Bio-pharm Technology Co., Ltd., Shanghai, China).

### 2.11. Statistical Analysis

The results were presented as the mean ± standard deviation. SPSS software (version 26.0, IBM, Chicago, IL, USA) was employed for statistical analysis, and Origin software (version 8.0, OriginLab Corp., Northampton, MA, USA) was utilized for graph generation. The initial three-way analysis of variances (ANOVAs) was conducted to assess the effects of packaging, electrostatic field, storage time, and their interactions. This initial model included storage time as a factor with five levels (0, 8, 16, 24, and 32 days). Since a significant interaction between packaging and storage time was identified (*p* < 0.05), the data from the storage period (days 8, 16, 24, and 32) were further analyzed using separate two-way ANOVAs (Packaging × EF) at each individual storage time point. Where the main effects or interactions were found to be significant in the two-way ANOVAs, Tukey’s Honestly Significant Difference test was used for post hoc multiple comparisons. The baseline data from day 0 (C0) were analyzed separately via one-way ANOVA. A significance level of *p* < 0.05 was applied for all tests.

UPARSE v7.1 software (http://drive5.com/uparse/, accessed on 5 April 2025) was used to cluster quality-filtered and merged sequences into operational taxonomic units (OTUs) at 97% sequence similarity and to remove chimeras. All data analyses were performed on the Majorbio I-Sanger Cloud Platform (https://cloud.majorbio.com, accessed on 10 April 2025). Specifically, alpha diversity indices (Chao1, Shannon) were calculated using Mothur software v1.48.0 (http://www.mothur.org/wiki/Calculators, accessed on 2 May 2025), and group differences in alpha diversity were assessed using the Wilcoxon rank-sum test. Principal coordinate analysis (PCoA) based on Bray–Curtis distances was used to examine the similarity of microbial community structure among samples. Spearman’s rank correlation analysis was used to select species for correlation network analysis.

## 3. Results and Discussion

### 3.1. WHC

**Figure 2:**
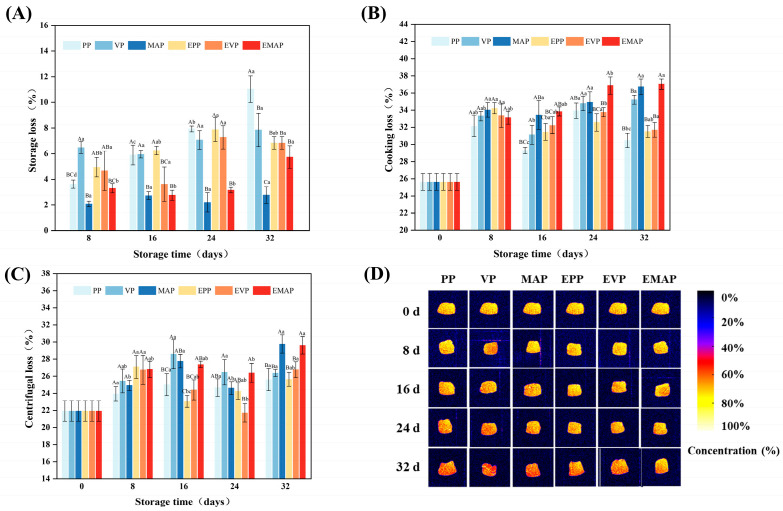
Changes in storage loss (**A**), cooking loss (**B**), centrifugal loss (**C**), and Proton density plot (**D**) of pork stored at −2.0 ± 0.5 °C for 32 days under different storage and packaging methods. a–d: Significant differences existed between the same treatment group on different storage days (*p* < 0.05). A–C: Significant differences existed between different treatment groups with the same storage days (*p* < 0.05). PP group: pork samples with polyethylene packaging; VP group: vacuum-packed pork samples; MAP group: modified atmosphere-packed pork samples; EPP group: PP treated under EF; EVP group: VP treated under EF; EMAP group: MAP treated under EF.

WHC is a crucial factor influencing meat quality, reflecting the ability of meat to retain moisture during transportation, storage, and processing. It can be evaluated by parameters such as storage loss, centrifugation loss, and cooking loss [9,25]. These indices are closely correlated with muscle protein characteristics, meat functionality, and processed product yield, playing a significant role in meat quality control. Figure 2A illustrates the impact of different treatments on the drip loss of pork. With the extension of storage time, the drip loss of pork in all groups showed an increasing trend. During controlled freezing-point storage, endogenous proteolytic enzyme systems degrade muscle proteins (MPs), leading to the disruption of their structure, reduced water binding capacity, and consequently, the migration of moisture from the interior to the exterior of the myofibrils, thereby increasing drip loss [21]. During the 16–32 days storage period, pork samples in the VP and MAP groups exhibited lower drip loss compared to the PP group, with the MAP group showing the most pronounced reduction (*p* < 0.05), indicating that MAP effectively inhibits drip loss. Notably, the presence or absence of the EF had no significant effect on storage loss during the initial storage period (8–24 days). However, at day 32, the storage loss in the PP group was significantly higher than that in the EPP group (*p* < 0.05), approximately 1.61 times greater, suggesting that the application of a weak electric field can significantly reduce the storage loss during long-term storage.

Figure 2B illustrates the effects of different treatments on pork cooking loss. Overall, cooking loss showed an increasing trend with prolonged storage time. PP packaging exhibits lower cooking losses throughout the entire storage period compared to MAP and VP methods, regardless of whether an EF is applied. This may be attributed to the removal of substantial exudate from PP packaged samples prior to cooking loss measurement [28]. However, no significant difference in cooking loss was observed between samples subjected to EF treatment and those without, both during the early storage phase (8–16 days) and at the later stage (32 days). This result is inconsistent with the findings reported by Xu et al. (2023), which may be primarily due to the relatively low EF intensity applied in the present study [9].

Figure 2C illustrates the effect of different treatments on the centrifugation loss of pork. As the controlled freezing-point storage time was extended, the centrifugation loss of the samples increased. Centrifugation loss is typically associated with protein structure [24,29]. Due to the denaturation of myosin, the internal water distribution of pork changes, leading to a significant decrease in free water content and a substantial increase in centrifugation loss with prolonged storage [30]. Between 16 and 24 days of controlled freezing-point storage, the centrifugation loss of meat samples from the EVP groups was significantly lower than that in the VP treatments (*p* < 0.05). The weak EF may promote the formation of hydrogen bond structures among water molecules in muscle, altering the structure of water molecules and their binding status with MPs, thereby reducing moisture loss during storage [21].

MRI, a non-invasive analytical technique, enables high-spatial-resolution mapping of water distribution within intact biological samples [31]. As shown in Figure 2D, T2-weighted MRI pseudo-color images of pork samples revealed distinct spatial heterogeneity in signal intensity. Fresh samples exhibited vivid yellow hues, indicative of higher free water content. With prolonged storage, signal intensity progressively diminished, manifested as a chromatic transition from bright yellow to intermixed yellow-red zones and ultimately to dominant red regions, correlating with moisture migration [32]. Comparative analysis of packaging modalities demonstrated that PP group samples displayed extensive red coloration after 32 days of storage, corresponding to severe moisture loss (storage loss: 11.03%). In contrast, EPP samples retained significantly larger yellow-red transitional zones, confirming enhanced water retention capacity under the weak EF (storage loss: 6.83%). This observation aligns with the storage loss (Figure 2A), corroborating a synergistic dielectric interaction between the EF and the packaging matrix, which significantly enhanced the responsiveness of PP packaging systems to electrostatic interventions during prolonged storage periods.

### 3.2. Changes in Color

**Figure 3:**
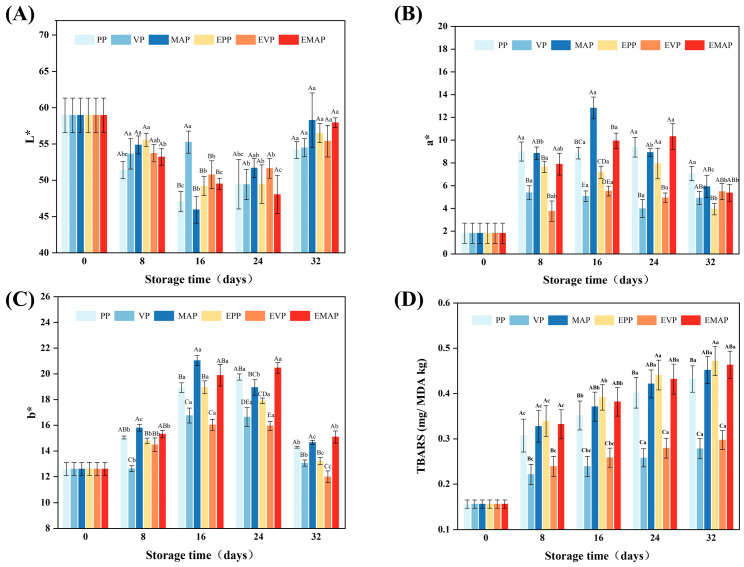
Changes in L* value (**A**), a* value (**B**), b* value (**C**), and TBARS (**D**) of pork stored at −2.0 ± 0.5 °C for 32 days under different storage and packaging methods. a–c: Significant differences existed between the same treatment group on different storage days (*p* < 0.05). A–E: Significant differences existed between different treatment groups with the same storage days (*p* < 0.05). PP group: pork samples with polyethylene packaging; VP group: vacuum-packed pork samples; MAP group: modified atmosphere-packed pork samples; EPP group: PP treated under EF; EVP group: VP treated under EF; EMAP group: MAP treated under EF.

Meat color is one of the most critical attributes determining consumer acceptance of fresh pork [33]. During controlled freezing-point storage, meat color stability is influenced by a complex interplay of factors, including myoglobin oxidation state, lipid oxidation, and microbial activity [34]. In this study, the color parameters (L*, a*, b*) of pork samples were measured during storage, and the results are shown in Figure 3A–C. The lightness (L* value) of all samples first decreased and then increased. The initial decrease may be related to slight changes in muscle surface WHC and alterations in myofibrillar structure, leading to changes in light scattering properties. The subsequent increase during later storage stages is typically attributed to water exudation and surface structural changes caused by the combined effects of protein denaturation, oxidation, and microbial metabolism, resulting in increased light reflectance. The a* (redness) and b* (yellowness) values generally exhibited an initial increase followed by a decrease. The initial increase in a* value may be due to the conversion of myoglobin to deoxymyoglobin, which is red, under low oxygen partial pressure [35]. The subsequent decrease directly reflects the irreversible oxidation of oxymyoglobin to brownish metmyoglobin. The packaging method had a significant impact on these changes (*p* < 0.05) [24]. The a* and b* values of the VP group were significantly lower than those of the MAP and PP groups throughout the storage period. This is mainly attributed to the fact that the vacuum environment greatly limits oxygen exposure, inhibiting the formation of bright red oxymyoglobin and resulting in a darker red color [36]. Furthermore, the hypoxic environment effectively retards oxygen-dependent lipid oxidation and the growth of aerobic microorganisms, which directly contributes to the significant decrease in b* value observed in the VP group [37]. After 24 days of storage, the b* value of the EPP group was lower than that of the PP group, although the difference was not statistically significant (*p* > 0.05). In contrast, the b* value of the EVP group was significantly lower than that of the VP group (*p* < 0.05). These results suggest that the application of an electric field differentially affects color changes in samples packaged using different methods, potentially due to the characteristics of the packaging materials.

### 3.3. Changes in TBARS

TBARS, recognized as a pivotal biomarker of lipid peroxidation, quantitatively reflects oxidative deterioration levels in meat matrices. This correlation establishes TBARS as an essential analytical parameter for evaluating meat quality degradation and spoilage mechanisms [38]. The determined TBARS values of pork samples across different treatment groups, as depicted in Figure 3D, consistently increased with extended storage time. Throughout storage, TBARS levels in most groups remained below 0.5 mg MDA/kg, indicating that the storage conditions effectively retarded lipid oxidation. This effect was most pronounced under VP, which suppressed TBARS accumulation more markedly than MAP or PP, resulting in consistently lower values. Notably, the TBARS increase in VP occurred at the slowest rate, with values remaining low (0.30 mg MDA/kg) even after 32 days. This is most plausibly attributed to the anaerobic environment established by VP, which effectively delays lipid oxidation [39]. Conversely, electric field treatment (3.2–3.3 kV/cm) did not produce statistically significant effects on TBARS values. In VP systems, the markedly restricted oxygen diffusion and the inherently low baseline lipid oxidation rate likely buffered the marginal effect of the electric field, preventing its manifestation as a detectable difference in TBARS. Although electric fields are theoretically posited to induce cell membrane rupture, potentially accelerating lipid oxidation and promoting the migration of pro-oxidants and unsaturated fatty acids in muscle tissue, our findings are consistent with existing literature. Previous studies reported no significant TBARS changes under similar electric field intensities (3.0 kV/cm for fresh turkey breast) [40]. Therefore, it is plausible that the EF application did not induce significant cell membrane damage, thereby preventing accelerated lipid oxidation.

### 3.4. pH

**Figure 4:**
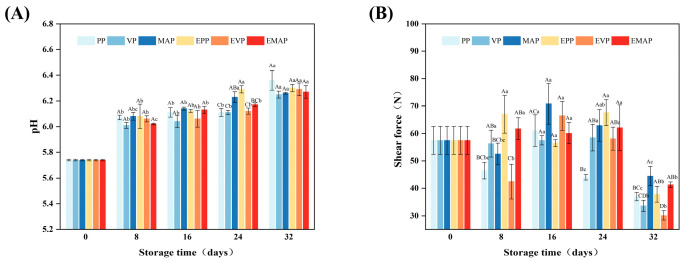
Changes in pH value (**A**) and shear force (**B**) of pork stored at −2.0 ± 0.5 °C for 32 days under different storage and packaging methods. a–c: Significant differences existed between the same treatment group on different storage days (*p* < 0.05). A–D: Significant differences existed between different treatment groups with the same storage days (*p* < 0.05). PP group: pork samples with polyethylene packaging; VP group: vacuum-packed pork samples; MAP group: modified atmosphere-packed pork samples; EPP group: PP treated under EF; EVP group: VP treated under EF; EMAP group: MAP treated under EF.

Figure 4A illustrates the temporal changes in pork pH values during controlled freezing-point storage under different treatments. Pork pH generally increased over the storage period, and the subsequent rise was mainly due to the accumulation of alkaline compounds such as ammonia, enzymatically derived amines, as well as the presence of spoilage microbes and protein decomposition [41,42]. Notably, neither packaging type (PP, VP, MAP) nor EF treatment significantly influenced pH changes (*p* > 0.05). This is likely because the controlled freezing-point conditions (−2.0 ± 0.5 °C) effectively inhibited microbial and enzymatic activities.

### 3.5. Shear Force

Pork tenderness, assessed via shear force, demonstrates a downward trend with prolonged storage, as depicted in Figure 4B. The tenderization of post-mortem muscle is a process driven by endogenous proteases, which degrade myofibrillar and cytoskeletal proteins, leading to a decrease in shear force [43]. By day 32 of storage, the MAP system exhibited a greater ability to retard meat sample tenderization compared to PP and VP systems. This observation is consistent with the results reported by Zakrys-Waliwander et al. (2010) [44] and may be attributed to the influence of the high-oxygen atmosphere on protein oxidation and cross-linking, which can modulate meat tenderness during storage [45]. Importantly, the application of an electric field did not negatively influence the tenderness of the samples.

### 3.6. Microbiological Analyses

**Figure 5:**
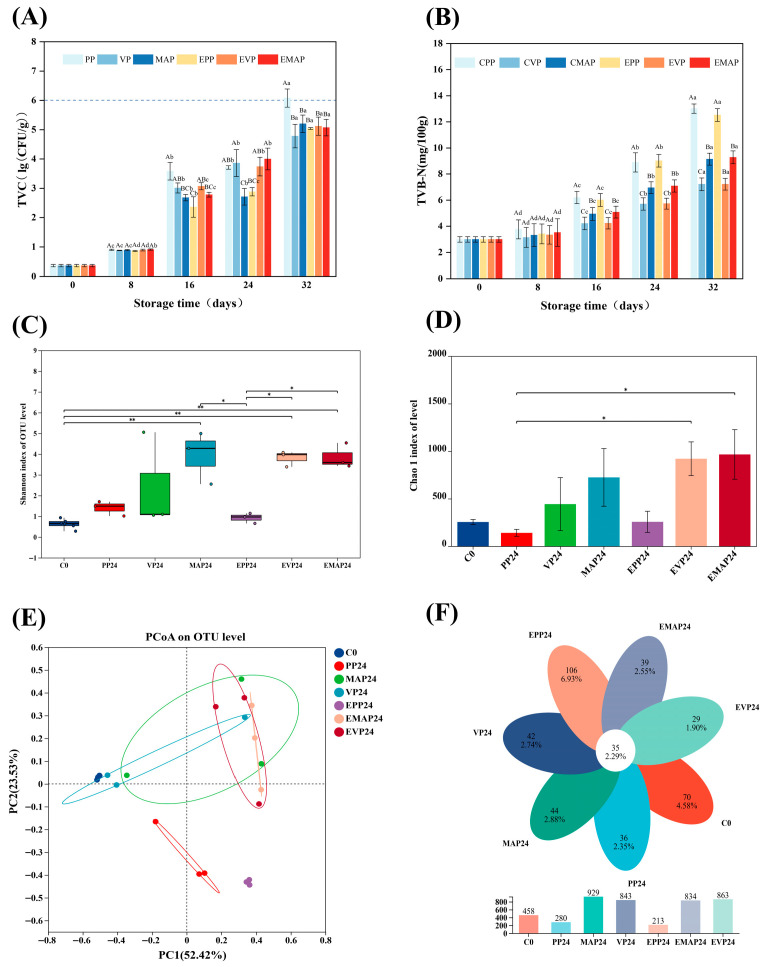
TVC (**A**), TVB-N (**B**), microbial α-diversity indices (Shannon index (**C**) and Chao1 (**D**)), principal coordinate analysis of microbial communities (**E**), and Venn diagram (**F**) in pork under different packaging conditions during controlled freezing-point storage (−2.0 ± 0.5 °C) at the genus level. a–d: Significant differences existed between the same treatment group on different storage days (*p* < 0.05). A–C: Significant differences existed between different treatment groups with the same storage days (*p* < 0.05). *: *p* < 0.05, indicating statistical significance; **:* p* < 0.01, indicating highly statistical significance. PP group: pork samples with polyethylene packaging; VP group: vacuum-packed pork samples; MAP group: modified atmosphere-packed pork samples; EPP group: PP treated under EF; EVP group: VP treated under EF; EMAP group: MAP treated under EF; C0: fresh pork sample; PP24: PP group sample stored for 24 days; VP24: VP group sample stored for 24 days; MAP24: MAP group sample stored for 24 days; EPP24: EPP group sample stored for 24 days; EVP24: EVP group sample stored for 24 days; EMAP24: EMAP group sample stored for 24 days; OTUs: operational taxonomic units.

Microbial contamination and subsequent growth are major factors contributing to spoilage in fresh meat [27]. The changes in TVC of each treatment group during controlled freezing-point storage are shown in Figure 5A. After 32 days of controlled freezing-point storage, the TVC of all samples increased from 0.37 lg (CFU/g) on day 0 to 4.78–6.08 lg (CFU/g). In the PP group, the TVC exceeded 6.00 lg (CFU/g) on day 32, indicating spoilage. In contrast, VP and MAP systems suppressed microbial growth, maintaining TVC below 6.00 lg (CFU/g) for at least 32 days. During controlled freezing-point storage from day 16 to day 32, the EPP group exhibited significantly lower TVC value compared to the PP group (*p* < 0.05). However, the application of the EF did not significantly influence the microbial counts in either the MAP or VP system. This could be attributed to the lower barrier properties of the PP system and to a potential response effect of the EF on samples in the PP group. Furthermore, the lower initial TVC also played a crucial role in delaying sample spoilage.

### 3.7. TVB-N

During storage, spoilage microorganisms and endogenous enzymes in pork contribute to the accumulation of alkaline nitrogenous substances, such as TVB-N. TVB-N directly indicates the extent of protein degradation and microbial spoilage, serving as a key indicator of pork freshness and quality deterioration [46]. According to Zhang et al. (2023), a TVB-N value below 15 mg/100 g was applied as a criterion for fresh meat [47]. Figure 5B illustrates the TVB-N trend during storage, which showed an initial slow change for 8 days, followed by a rapid increase over time. However, TVB-N values remained below 15 mg/100 g throughout storage, which can be attributed to the inhibitory effect of the low storage temperature. Furthermore, after 16 days, the VP and EVP groups consistently exhibited lower TVB-N values than the other groups (*p* < 0.05), although no significant differences were observed between VP and EVP themselves. This finding is consistent with the TBARS results.

### 3.8. Microbial Abundance and Diversity

To elucidate how microbial diversity and abundance change under the combined effects of an EF and different packaging conditions during controlled freezing-point storage, high-throughput sequencing of the bacterial communities in pork was conducted using the Illumina MiSeq platform. The alpha diversity for all samples was assessed using the Chao1 and Shannon indices, as shown in Figure 5C,D. The Chao1 index was used to estimate the richness or abundance of microbial species present in each community [48]. The Shannon index was used to characterize the diversity of each microbial community [49]. The C0 group had the lowest Shannon index (*p* < 0.05), whereas extended storage durations resulted in higher values. This finding suggests that prolonged storage increases microbial community diversity in pork, independent of packaging. On the 24th day of storage, EMAP and EVP groups exhibited higher Chao1 and Shannon indices compared to the PP group.

To evaluate beta diversity, which represents the differences in species composition between samples, PCoA was employed. This analysis revealed distinct clustering patterns, with the first two principal components explaining 52.42% and 23.53% of the data variance, respectively, and accounting for a cumulative 75.95% of the total variation (Figure 5E). The VP group exhibited the most similar bacterial structure to fresh pork, as indicated by its close proximity to the fresh pork samples on the PCoA plot. The marked separation of the PP and EPP samples from the remaining samples underscores the significant influence of packaging on the microbial community structure. The Venn diagram illustrates the distribution of shared and unique operational taxonomic units (OTUs) among the different sample groups. As shown in Figure 5F, 35 OTUs were shared among all samples at both the initial storage time and at day 24. The numbers of unique genera in the C0, PP24, VP24, MAP24, EPP24, EVP24, and EMAP24 groups were 70, 36, 42, 44, 106, 29, and 39, respectively, with PP24 and EPP24 exhibiting a higher number of unique genera, suggesting a potential influence of these treatments on the microbial community. Furthermore, compared to the initial storage time, the VP and MAP packaging methods resulted in a greater number of OTUs at day 24 of storage than the PP packaging method, highlighting the impact of packaging on microbial diversity. The lower OTU abundance in cling film packaging compared to VP and MAP groups on day 24 of storage is reasonably explained by multiple contributing factors. Firstly, cling film’s high permeability and moisture evaporation likely inhibit moisture- and oxygen-sensitive microbes due to surface drying and altered oxygen exposure, reducing detectable OTUs. Secondly, VP and MAP create a low-oxygen, high-CO_2_ environment that selectively favors more tolerant microbes, allowing their co-existence and thus maintaining or increasing OTU abundance, despite inhibiting some aerobic bacteria. Collectively, different packaging methods employ distinct microbial selection mechanisms, significantly altering the microbial ecological dynamics during meat storage.

**Figure 6:**
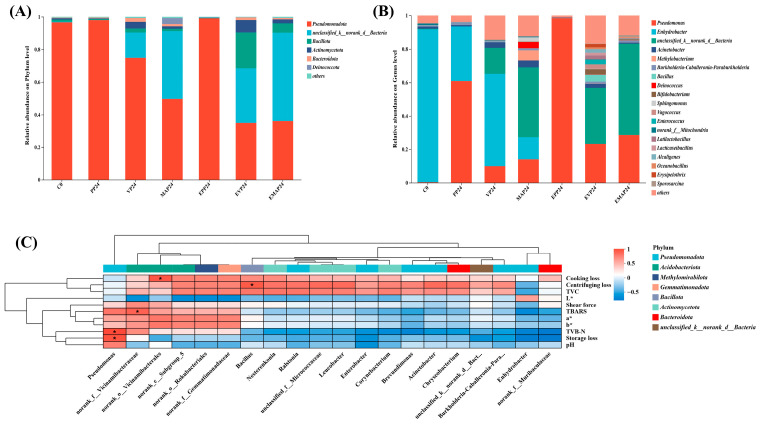
Relative abundance of microbial communities at the phylum (**A**) and genus (**B**) levels, and spearman correlation heatmap of pork physicochemical properties (cooking loss, centrifugation loss, TVC, L*, shear force, TBARS, a*, b*, TVB-N, storage loss, pH) and dominant bacteria (**C**) during controlled freezing-point storage (−2.0 ± 0.5 °C) under different packaging conditions. Spearman’s r: −1 to 1; r < 0 (negative), r > 0 (positive). * *p* ≤ 0.05. PP group: pork samples with polyethylene packaging; VP group: vacuum-packed pork samples; MAP group: modified atmosphere-packed pork samples; EPP group: PP treated under EF; EVP group: VP treated under EF; EMAP group: MAP treated under EF; C0: fresh pork sample; PP24: PP group sample stored for 24 days; VP24: VP group sample stored for 24 days; MAP24: MAP group sample stored for 24 days; EPP24: EPP group sample stored for 24 days; EVP24: EVP group sample stored for 24 days; EMAP24: EMAP group sample stored for 24 days.

Figure 6A,B illustrate the relative abundances of dominant microbial phyla and genera in pork during controlled freezing-point storage. Across the storage period, *Pseudomonadota*, *Bacillota*, *Actinomycetota*, and *Bacteroidota* constituted the major phyla (Figure 6A). Notably, in the PP24 group, *Pseudomonadota* was overwhelmingly dominant, comprising nearly 90% of the relative abundance. Conversely, the VP24 group exhibited a significant decrease in *Pseudomonadota* relative abundance, accompanied by an increase in unclassified_k__norank_d__Bacteria and *Bacillota*. This indicates that the VP system may inhibit the growth of *Pseudomonadota* while favoring the proliferation of other bacterial taxa. The microbial community structure of the MAP24 group resembled that of VP24, except for a higher relative abundance of *Bacillota*, which is potentially attributable to the specific gas composition of the modified atmosphere. EF treatment effects on microbial community structure varied with the packaging method. The EPP24 group showed a slight increase in the relative abundance of the dominant phylum *Pseudomonadota* compared with PP24, with *Pseudomonadota* remaining dominant. In the EVP24 group, the relative abundance of *Bacillota* was significantly higher than in the VP24 group. This suggests that EF treatment may enhance the inhibitory effect of VP on *Pseudomonadota* while promoting the growth of unclassified_k__norank_d__Bacteria. The EMAP24 group exhibited the most complex microbial community, with the lowest relative abundance of *Pseudomonadota* and increased relative abundances of *Bacillota*, *Actinomycetota*, and *Bacteroidota*, indicating that the combination of EF treatment with MAP may drive diversification of the microbial community.

At the genus level, *Enhydrobacter*, *Acinetobacter*, and *Methylobacterium* were predominant (Figure 6B). These bacteria mainly originate from meat processing environments, such as soil or water [50,51]. Extended controlled freezing-point storage markedly shifted the microbial composition: *Enhydrobacter* abundance decreased, whereas *Pseudomonas*, unclassified_k__norank_d__Bacteria, and *Deinococcus* became more prevalent. As a typical aerobic spoilage genus, *Pseudomonas* reached its highest relative abundance (approximately 60%) in the PP group, a finding directly linked to the high oxygen permeability of this packaging material. In contrast, VP (VP/EVP) and MAP (MAP/EMAP) packaging significantly reduced the Pseudomonas proportion (VP24: 10%; EVP24: 25%; MAP24: 15%; EMAP24: 30%), demonstrating their superior efficacy, particularly when combined with EF treatment, in suppressing this spoilage organism. In addition, EF treatment reduced the proportion of *Acinetobacter*. In summary, the combination of an EF with vacuum or MAP can favorably modulate the pork storage microbial community by suppressing dominant spoilage bacteria and maintaining microbial balance.

### 3.9. Correlation Between Microorganisms and Physiochemical Characteristics

This study employed Spearman’s correlation analysis to investigate the relationships between dominant bacterial communities and key physicochemical characteristics in pork samples treated with EF combined with different packaging methods under controlled freezing-point storage conditions. In Figure 6C, red hues indicate positive correlations, where deeper coloration represents stronger positive associations (correlation coefficients approaching +1). Conversely, blue hues denote negative correlations, with darker shades corresponding to stronger negative relationships (correlation coefficients closer to −1).

As illustrated in the Spearman correlation heatmap (Figure 6C), significant correlations were identified between specific bacterial taxa and the physicochemical deterioration of pork samples. Notably, at the genus level, *Pseudomonas* exhibited a significant positive correlation with TVB-N and storage loss (*p* < 0.05). This strong association can be attributed to the metabolic activities of *Pseudomonads* [2]. As efficient spoilage organisms, *Pseudomonas* species proliferate by utilizing glucose and amino acids in meat, leading to the production of volatile compounds such as ammonia, amines, and sulfides, which directly contribute to the increase in TVB-N, a key indicator of protein decomposition [11,52]. Concurrently, their secretion of extracellular enzymes, including proteases and lipases, disrupts the muscle tissue integrity, compromising the WHC and thereby resulting in increased purge or storage loss.

The effectiveness of different packaging methods and EF treatments in modulating these correlations is evident. VP and MAP, often combined with EF treatment (EVP and EMAP), significantly reduced the relative abundance of *Pseudomonadota* and consequently mitigated the increase in TVB-N and storage loss compared to PP. Although *Pseudomonadota* abundance remained higher in EPP24 compared to PP24, the combined treatments of EVP24 and EMAP24 demonstrated synergistic effects in suppressing *Pseudomonadota* and promoting microbial community diversity. Compared to the control groups (Figure 6A), EVP24 and EMAP24 treatments significantly reduced *Pseudomonadota* abundance (particularly in EVP24) and increased the abundance of *Bacillota* and *Actinomycetota* (particularly in VP24).

Furthermore, other significant correlations underscore the complex role of the microbiota in quality decline. The significant positive correlation between *norank_f__Vicinamibacteraceae* and TBARS (*p* < 0.05) suggests a potential, though less defined, role of this taxon in promoting lipid oxidation. Similarly, the significant positive correlation of *norank_o__Vicinamibacterales* with cooking loss and of *Bacillus* with centrifuging loss (*p* < 0.05) indicates that these bacteria are also involved in the deterioration of WHC. Among these, the pervasive and strong correlations of *Pseudomonas* with multiple quality parameters highlight their pivotal role as a primary contributor to quality deterioration during storage. The increase in storage loss, in particular, is a direct consequence of the structural damage to the meat matrix inflicted by *Pseudomonas* metabolism.

## 4. Conclusions

This study elucidates the synergistic preservation effects of the EF combined with various packaging methods on fresh pork during controlled freezing-point storage. The foundational roles of VP and MAP were confirmed to maintain color, delay lipid oxidation (e.g., TBARS in VP remained at 0.30 mg MDA/kg on day 32), and inhibit microbial growth (maintaining TVC below 6.00 lg CFU/g for over 32 days). The application of an EF further enhanced preservation, primarily by modulating the microbial ecology in a packaging-dependent manner. In the O_2_ permeability PP system, EF significantly reduced storage loss from 11.03% to 6.83% after 32 days. More importantly, when integrated with VP or MAP, EF effectively suppressed dominant spoilage bacteria, notably reducing the relative abundance of *Pseudomonas* to 25% (EVP24) and 30% (EMAP24), respectively, compared to 60% in PP24. Spearman correlation analysis mechanistically linked the EF-driven microbial shifts to quality preservation, revealing a significant positive correlation between *Pseudomonas* abundance and the increases in TVB-N and storage loss. In contrast, the impact of EF on lipid oxidation and pH was negligible, likely due to the dominant inhibitory effect of the low storage temperature. In conclusion, integrating EF technology with VP or MAP packaging presents a promising, chemical-free strategy for shelf-life extension, achieved through direct microbial inhibition and indirect quality maintenance via community modulation.

Future research should focus on optimizing EF parameters (e.g., intensity, frequency, timing) and packaging gas compositions to maximize this synergy. Furthermore, employing metabolomic and metatranscriptomic analyses is recommended to elucidate the functional changes within the microbial community and their direct metabolic impact on meat quality deterioration.

## Data Availability

The original contributions presented in this study are included in the article. Further inquiries can be directed to the corresponding authors.

## Abbreviations

The following abbreviations are used in this manuscript:

EF	Electrostatic Field
HVEF	High-Voltage Electrostatic Field
PP	Polyethylene Packaging
VP	Vacuum Packaging
MAP	Modified Atmosphere Packaging
EPP	Electrostatic Field Treatment for PP
EVP	Electrostatic Field Treatment for VP
EMAP	Electrostatic Field Treatment for MAP
TBARS	Thiobarbituric Acid Reactive Substances
WHC	Water-Holding Capacity
TVC	Total Viable Counts
TVB-N	Total Volatile Basic Nitrogen
MRI	Magnetic Resonance Imaging
PCoA	Principal Coordinate Analysis
ANOVAs	Analysis of Variances
OTUs	Operational Taxonomic Units
